# Supplementary material for: In Vitro Skin Retention of Crisaborole after Topical Application
Source: Pharmaceutics. 2020 May 28;12(6):491. doi: 10.3390/pharmaceutics12060491 (PMC7355453; doi:10.3390/pharmaceutics12060491)
Supplement: Supplementary file 1 [file pharmaceutics-12-00491-s001.pdf]

pharmaceutics

Supplementary Materials: In Vitro Skin Retention of Crisaborole after Topical Application

Adriana Fantini, Anna Demurtas, Sara Nicoli, Cristina Padula, Silvia Pescina and Patrizia Santi

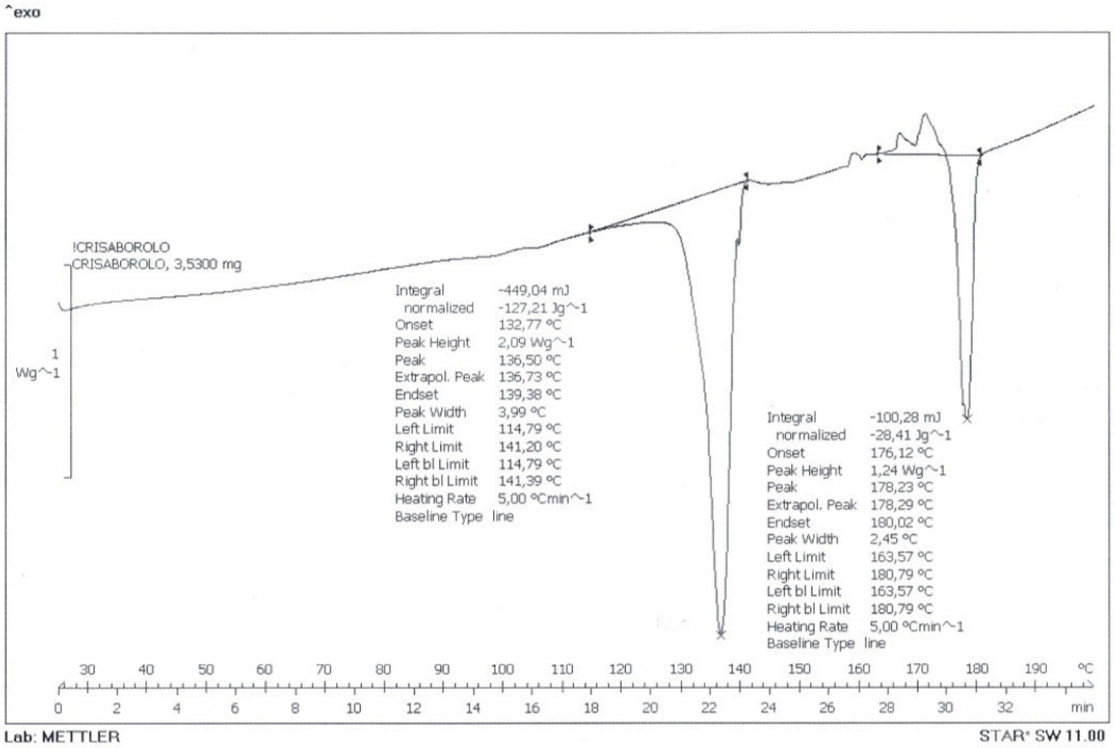

Figure S1. trace of crisaborole (heating rate at 5 °C/min), recorded under a flux of dry nitrogen (100 mL/min).

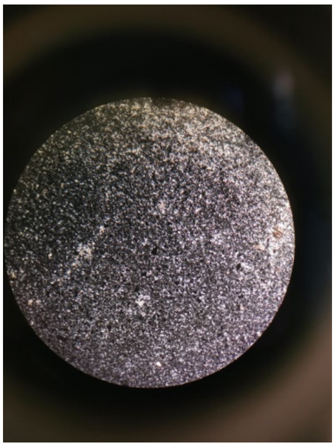

Figure S2. Polarized-light microscopy (10X) image of the prepared ointment".

© 2020 by the authors. Submitted for possible open access publication under the terms and conditions of the Creative Commons Attribution (CC BY) license (http://creativecommons.org/licenses/by/4.0/).
